# Fertility desire and associated factors among people living with HIV in Ethiopia: a systematic review and meta-analysis

**DOI:** 10.1186/s13690-020-00504-0

**Published:** 2020-11-23

**Authors:** Getu Mosisa, Reta Tsegaye, Bizuneh Wakuma, Diriba Mulisa, Werku Etefa, Muktar Abadiga, Ginenus Fekadu, Adugna Oluma, Ebisa Turi

**Affiliations:** 1 School of Nursing and Midwifery, Institute of Health Sciences, Wollega University, Nekemte, Ethiopia; 2 Department of Public health, Institute of Health Sciences, Wollega University, Nekemte, Ethiopia; 3 Department of Pharmacy, Institute of Health Sciences, Wollega University, Nekemte, Ethiopia

**Keywords:** ART, Fertility desire, People living with HIV, Meta-analysis, Ethiopia

## Abstract

**Background:**

Increased Anti-Retroviral Therapy (ART) coverage improves health status and the survival of people living with Human Immunodeficiency Virus (HIV) as a result, reproductive health needs of the clients are increased. As part of continuum HIV care, understanding fertility desire and reproductive health care needs of HIV positive peoples will play paramount role in planning and delivering appropriate health services. The finding of studies conducted on the fertility desire and associated factors among People Living with HIV in Ethiopia presented inconclusive. Therefore, this study aimed to assess the pooled prevalence of fertility desire and associated factors among people living with HIV in Ethiopia.

**Methods:**

A total of 26 studies conducted in Ethiopia were included in this Meta-analysis. Pub Med, HINARI, Google scholar and Google data bases were searched. Data from the included articles were extracted using a standardized data extraction tool. The included studies were analyzed using a random effects meta-analysis model. Analysis was done Using STATA version 14 statistical software. Heterogeneity was assessed statistically using the standard Chi-square, I^2.^ The association between fertility desire and factors were examined using a random effects model.

**Result:**

In this meta-analysis, the pooled prevalence of fertility desire in Ethiopia is 42.21% (95%CI 39.18, 45.25). Fertility desire is significantly associated with sex: being female (OR = 0.71,95%CI 0.57,0.86), partners desire (OR = 16.8, 95% CI: 9.45, 29.88), not having child (OR = 5.46 95%CI 4.24, 7.040), age < 30(OR = 2.34, 95%CI 2.10, 2.60), formal education (OR = 1.31 95%CI 1.09, 1.59)). However, use of family planning, residence, and Knowledge on Prevention of Mother to Child Transmission and disclosure status didn’t show association with fertility desire.

**Conclusion:**

In this finding, significant people of living with HIV have a desire to have a child. The finding showed the need to strengthen fertility desire and reproductive health care needs of HIV positive peoples. Therefore, strengthening the integration of fertility related issues with HIV continuum care will play a paramount role in averting risky sexual behaviors and Prevention of Mother to Child Transmission among peoples on ART.

## Background

Human Immunodeficiency Virus (HIV) remains the major public problem in the world. Globally, at the end of 2018, about 37.9 million people were living with HIV. About 1.7 million people became newly infected with HIV, 770000 people died from Acquired Immune Deficiency Syndromes (AIDS)-related illnesses. At the end of June 2019, 24.5 million people were accessing antiretroviral therapy. Every week around 6000 young women aged 15–24 years become infected with HIV. In sub-Saharan Africa, four in five new infections among adolescents aged 15–19 years are in girls. In 2013, in Ethiopia there were an estimated 793,700 people living with HIV [1, 2].

Globally, people living with HIV particularly, women of reproductive age group remained the highest share holder of HIV/AIDS related morbidity and mortality [3]. According to 2017 WHO report, in Ethiopia, the estimated number of women age greater than 15 years living with HIV was around 400,000 and the rate of mother to child transmission (MTCT) of HIV was 16 in 2016 [4].

Since the beginning of PMTCT, Ethiopia has implemented various policies and programs of PMTCT. For instance, In Ethiopia, the first national PMTCT guideline was developed in 2001. Following this further adjustments were made in the year 2007, 2011 and 2013 to include international recommendations [5]. The current PMTCT guideline (2013) is based on the World Health Organizations option B+ PMTCT protocol [6], in which all HIV-positive pregnant women are immediately offered antiretroviral therapy (ART) for life irrespective of their CD4 count. This in turn changed the life expectancy and quality of life for mothers, children, and their sexual partners more than ever before. Despite this, retention on PMTCT and fertility issues are becoming challenging among peoples on ART in Ethiopia [7]. Moreover, the integration of family planning along the HIV/AIDS care continuum is very poor in deed even though there is high unmet need for family planning [8].

Even though it is possible to have a healthy pregnancy and HIV-negative child, in low income country where there is low coverage of prevention of mother to child transmission and antiretroviral therapy (ART) is existed, the desire to have a child among people living with HIV remains a major public health problem [9].

Increased ART coverage improves health status and increase the survival of people living with HIV; as a result, reproductive health needs of the clients are increased. Today, peoples living with HIV/AIDS are living their sexual and reproductive life better than ever before; however, initiating ART and improved health status didn’t increase the fertility desire of PLHIV. The reproductive health needs, particularly fertility desire, family planning and pregnancy needs are complicated and decreased because of their HIV status. Their HIV status hinders the desire of the women to have more children. Therefore, focusing on the essential needs of PLHIV specifically, sexual health needs of HIV-positive people are essential to global HIV prevention [10–13].

Similar to HIV negative peoples, People living with HIV have strong fertility desire. But the fertility desire of PLHIV is lower than the general populations as a result of hindering factors [14, 15]. Studies have shown that factors such as age, gender, marital status, number of children, partner’s fertility desire, and HIV status of partner had an association with fertility desire of People living with HIV [16–18].

The finding of studies conducted on the fertility desire and associated factors among PLWH in Ethiopia presented wide ranging variations. For instance, the prevalence of fertility desire among PLWH in Ethiopia ranges from 23.58 to 66.12%. Beside to this there is no nationally pooled data regarding the fertility desire of PLWH in Ethiopia. Hence, this systematic review and meta-analysis was aimed to do the pooled prevalence of fertility desire and effect size of associated factors among people living with HIV in Ethiopia.

## Methods

### Search strategies

The search strategy aimed to find both published and unpublished studies. A three-step search strategy was utilized in this review. An initial limited search of Pub Med, Google Scholar, was undertaken followed by an analysis of the text words contained in the title and abstract, and of the index terms used to describe the article. A second search using all identified keywords and index terms was undertaken across all included databases. Thirdly, the reference list of all identified reports and articles was searched for additional studies. We searched databases include: Pub Med, HINARI, Google scholar and Google The search for unpublished studies such as thesis, and dissertations were checked in Addis Ababa University Digital Library. The literature search was done from November 10 to December 25, 2019. The search was carried out using the following keywords by Boolean operator: “fertility desire” OR “fertility intention” “OR” “fertility need” OR “desire to have child” AND “HIV patients” OR “PLHIV” OR “PLWHA” AND “Ethiopia”. The review protocol was registered in the International Prospective Register of Systematic Reviews (PROSPERO) (Registration number CRD42019130966) Availablefrom: https://www.crd.york.ac.uk/prospero/display_record.php?ID=CRD42019130966

### Selection and eligibility criteria

#### Inclusion criteria

This systematic review and meta-analysis included studies that were conducted on the prevalence and determinants of fertility desire among Peoples living with HIV/AIDS (PLWH) in Ethiopia. Two investigators (GM & DM) independently reviewed the contents of each articles and finally articles fulfilled the following criteria were included in the study. The Study populations were all people living with HIV/AIDS in Ethiopia. Studies, which were conducted in Ethiopia, were included in the review. For this review, we included all observational study designs reporting prevalence of fertility desire among PLHIV in Ethiopia. Articles reported in the English language were included.

#### Exclusion criteria

For this review, we excluded papers that were not fully accessible, after at least two email contact attempts with the primary authors.

### Outcome measurement

The review considered two main outcomes. Prevalence of Fertility desire (defined here as the desire to have child/children in the future) among people living with HIV was the primary outcome of this study. The second outcome of this study was to identify factors associated with fertility desire among PLHIV. For the second outcome, we determined the association between Fertility desire and associated factors in the form of the log odds ratio. Factors included in this review were sex, age, educational status, residence, use of family planning, Knowledge on PMTC, number of alive child, partner fertility desire and disclosure status.

### Data extraction

Two authors (GM and DM) extracted the data using data extraction checklists on Microsoft excel. For the outcome variable (fertility desire) and its determinants, data were extracted in a format of two by two tables, and then the log OR was calculated based on the findings of the original studies. The checklist for data extraction contains the title, author name, year of publication, region (the area where the study was conducted), study design, sample size, response rate, and the number of participants with the outcome. When articles did not have adequate data, the corresponding authors of the research articles were contacted through their email.

### Quality assessment

The quality of each original study was assessed using standardized critical appraisal instruments from the Joanna Briggs Institute Meta-Analysis of Statistics Assessment and Review Instrument [19]. In this review risk of bias was assessed using PRISMA guidelines with the two reviewers independently. Any disagreements between the two reviewers were addressed by taking the mean score of the two reviewers.

### Statistical analysis

The included studies were analyzed using a random effects meta-analysis model. Analysis was done Using STATA version 14 statistical software. Heterogeneity was assessed statistically using the standard Chi-square, I^2^and also explored using subgroup analyses based on the different quantitative study designs included in this review. Tables and figures were used as appropriate to aid in data presentation. Statistical significance was considered at *P* –value < 0.05. In this Meta regression for assess presence of publication bias, funnel plot and Egger test at 5% significant level were computed.

## Results

### Study selection

A systematic search of electronic databases and library catalogs identified a total of 62 articles. From these, 27 duplicates papers were removed. The abstracts and full text of the remaining 35 studies were assessed and screened for eligibility criteria based on the outcome variable. Then 9 articles were excluded for not meeting the inclusion criteria.

Finally, 26 articles that scored seven and above on the JBI quality appraisal eligibility criteria were included in the systematic review and meta-analysis. Preferred Reporting Items for Systematic Reviews and Meta-Analyses (PRISMA) flow diagram were used to present the systematic review process.

**Fig. 1:**
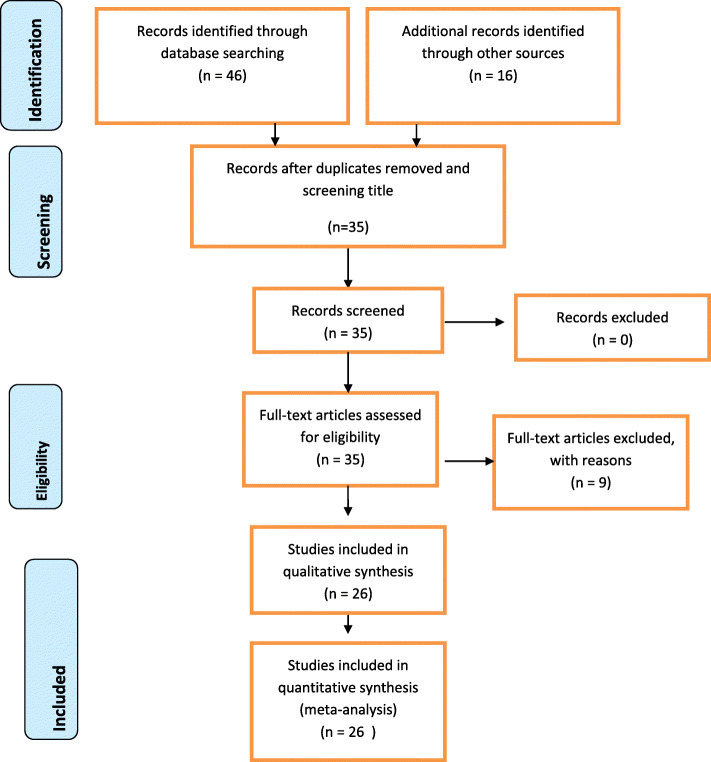
PRISMA flow diagram of included studies for the systematic review and meta-analysis of Fertility desire and associated factors among people living with HIV in Ethiopia, 2020

Twenty-six articles have been included in this Meta regression (Fig. 1) to show the pooled magnitude of fertility desire among peoples living with HIV in Ethiopia [20–45]. In those previous articles as individual record the smallest magnitude is finding in study conducted in Oromia regional state 23.58%(95%CI 20.27,26.89) [33] while the largest study is study conducted in Tigray regional state of Ethiopia 66.12% (95%CI 60.83, 71.42) [32] (Table 1).

**Table 1:** Summary of included Studies and prevalence of fertility desire among HIV positive peoples in Ethiopia, 2020

Author	Year of publication	Region	Study design	sample size	Prevalence(95%CI)
Ahmed et al. [20]	2014	Amhara	Cross-sectional	392	39.54 (34.70,44.38)
Haile, et al. [21]	2014	Harari	Cross-sectional	518	56.18 (51.90,60.45)
Gayessa et al. [22]	2013	Oromia	Cross-sectional	321	46.42 (40.96,51.87)
Abebe M et al. [23]	2015	Oromia	Cross-sectional	462	41.13 (36.64,45.61)
Kebede Y et al. [24]	2019	Oromia	Cross-sectional	242	39.26 (33.10,45.41)
Asfaw & Gashe [25]	2014	Addis Ababa	Cross-sectional	1855	43.94 (41.68,46.19)
Regassa T. etal [26]	2012	Oromia	Cross-sectional	592	35.64 (31.78,39.50)
Abebe M etal [27]	2012	SNNP	Cross-sectional	321	36.45 (31.18,41.71)
Melka et al. [28]	2014	Oromia	Cross-sectional	456	42.11 (37.57,46.64)
Aderajaw M [29].	2010	Amhara	Cross-sectional	1287	37.30 (34.65,39.94)
HailuChora et al. [30]	2017	SNNP	Cross-sectional	410	42.93 (38.14,47.72)
Amanuel Desta [31]	2009	Tigray	Cross-sectional	367	39.24 (34.24,44.23)
Alemayehu B et al. [32]	2012	Tigray	Cross-sectional	307	66.12 (60.83,71.42)
Hassen K et al. [33]	2013	Oromia	Cross-sectional	632	23.58 (20.27,26.89)
Mokwena K et al. [34]	2017	Oromia	Cross-sectional	362	43.65 (38.54,48.76)
Adilo et al [35]	2017	Addis Ababa	Cross-sectional	441	51.47 (46.81,56.14)
Mekonnen B et al. [36]	2019	Amhara	Cross-sectional	427	40.28 (35.63,44.93)
wondwosen et al. [37]	2019	Oromia	Cross-sectional	416	46.15 (41.36,50.94
Tedesse L. [38]	2011	Oromia	Cross-sectional	384	34.11 (29.37,38.86)
Demissie et al. [39]	2014	Oromia	Cross-sectional	340	39.12 (33.93,44.30)
Melaku etal [40]	2014	Tigray	Cross-sectional	964	45.54 (42.40,48.68)
Abbawa et al. [41]	2015	Amhara	Cross-sectional	422	33.41 (28.91,37.91)
Shiferaw et al. [42]	2019	Oromia	Cross-sectional	374	46.79 (41.73,51.85)
Tamene W et al. [43]	2007	Addis Ababa	Cross-sectional	460	40.22 (35.74,44.70)
Tesfaye et al. [44]	2012	Amhara	Cross-sectional	389	42.16 (37.25,47.07)
Gosa T [45].	2010	Addis Ababa	Cross-sectional	444	46.17 (41.53,50.81)

**Fig. 2:**
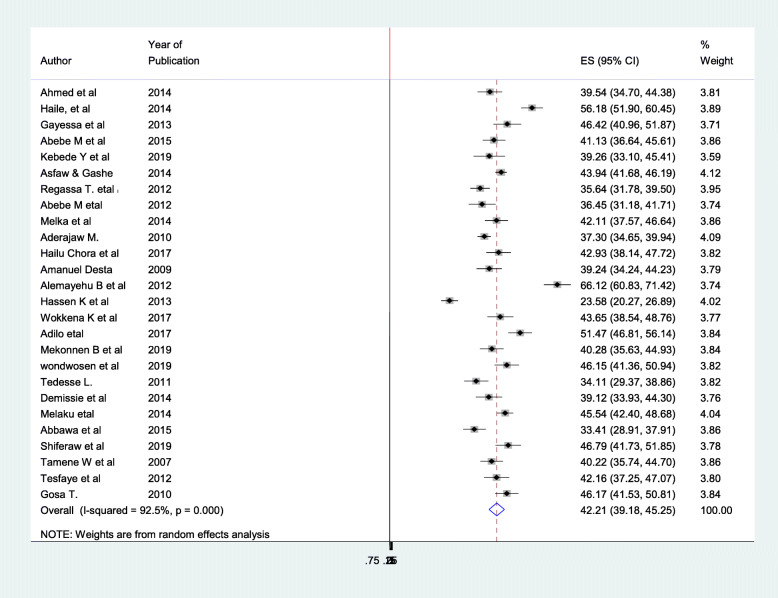
Forest plot of the pooled Prevalence of fertility desire among HIV positive peoples in Ethiopia, 2020

There is high heterogeneity in the study (I^2^ = 92.5%, *P* = 0.00) and due to this random effect model was used to find the pooled prevalence of fertility desire among peoples living with HIV aids in Ethiopia. Accordingly, the pooled Meta regression indicated the prevalence of fertility desire in Ethiopia is 42.21% (95%CI 39.18, 45.25) (Fig. 2).

### Subgroup analysis and publication bias

**Fig. 3:**
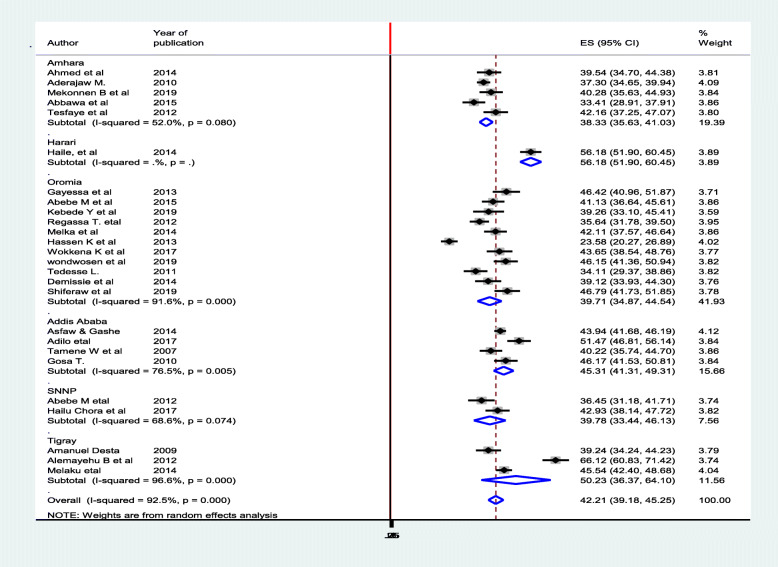
Sub-group analysis of prevalence of fertility desire among HIV positive peoples in Ethiopia, 2020

**Fig. 4:**
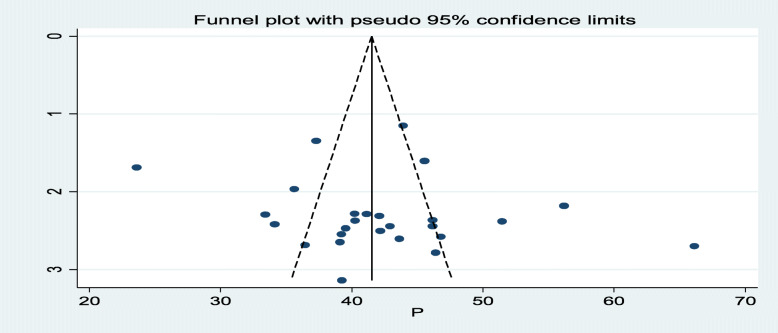
Funnel plot with 95% confidence limit of the pooled Prevalence of fertility desire among HIV positive peoples in Ethiopia, 2020

In this meta-analysis the prevalence of fertility desire among HIV positive peoples in Ethiopia have been computed subgroup analysis by region. Accordingly, the smallest magnitude of prevalence is observed in study conducted in Amhara regional state of Ethiopia 38.33% (95% CI 5.63, 41.03) [20, 29, 36, 41, 44]. The largest prevalence is observed in Harari regional state of Ethiopia that is 56.18(95% CI 51.90, 60.45) [21]. To assess publication bias, a graphical funnel plot and Egger’s test at 5% significance level were computed. The funnel plot shown a shape of symmetry, and Egger’s test was insignificance for the presence of publication (*P* = 0.362) (Figs. 3, 4).

### Statistical analysis and synthesis

**Fig. 5:**
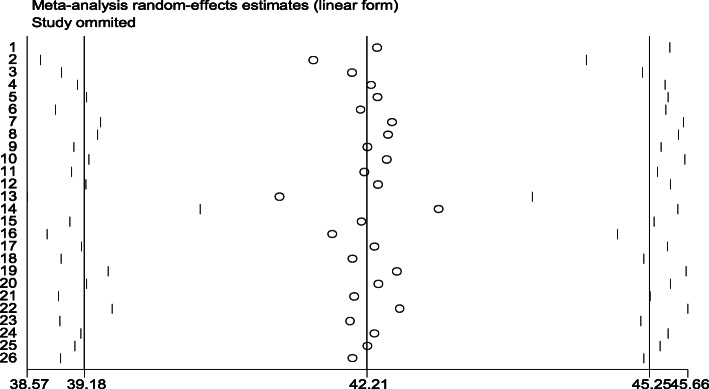
Sensitivity analysis for a single study influence on the overall study of fertility desire in Ethiopia, 2020

Sensitivity analysis was done using a random effect models to classify a single study influence on the general meta-analysis, and the result disclosed that there was no strong indication for the effect of the single study on the overall meta-analysis result (Fig. 5).

### Factors associated with fertility desire among HIV positive patients in Ethiopia

#### Association between fertility desire and sex

**Fig. 6:**
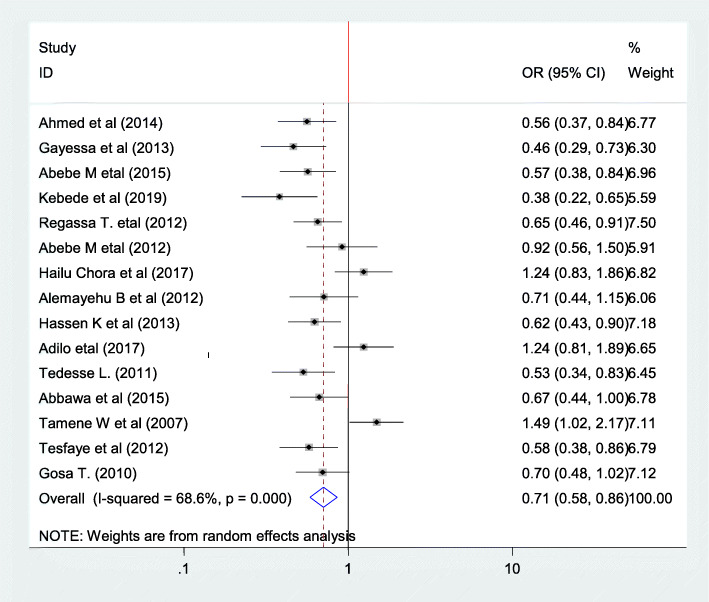
Forest plot of association between fertility desire among people with HIV positive and sex of the participants in Ethiopia, 2020

To see association between fertility desire and sex fifteen articles have been involved in this study [20, 22–24, 26, 27, 30, 32, 33, 35, 38, 41, 43–45]. From those fifteen articles nine of them showed significant association [20, 22–24, 26, 33, 38, 43, 44]. Six of the articles don’t show significant association [27, 30, 32, 35, 41, 45]. However, the pooled meta regression indicated that sex of the participant is associated with fertility desire that is female has 0.71 less likely have fertility desire as compared with men (female have 29% of wish to have fertility desire (OR = 0.71, 95%CI 0.57,0.86)). In this meta regression since there is moderate hetrogenicity random effect model was used(I^2^ = 68.6% *P* = 0.00) (Fig. 6).

#### Association between fertility desire and partner fertility desire

**Fig. 7:**
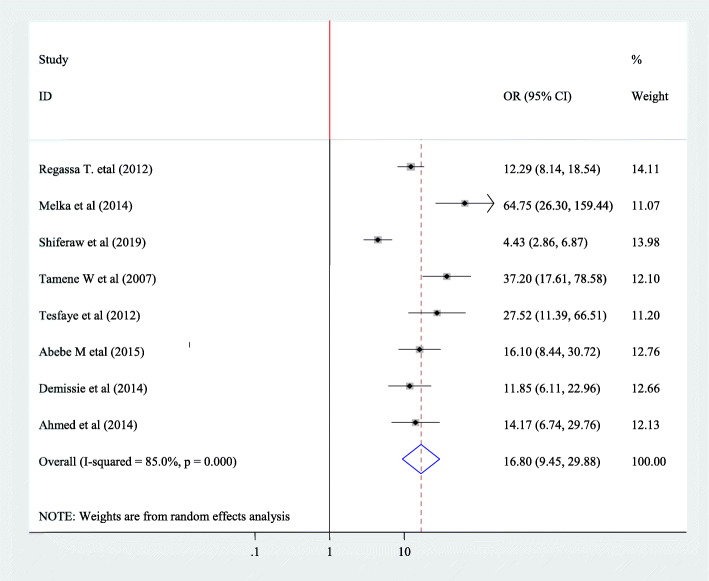
Forest plot of association between fertility desire among people with HIV positive and Partner fertility desire of the participants in Ethiopia, 2020

In this Meta regression, to find association between fertility desire and partner fertility desire, eight articles have been included [20, 23, 26, 28, 39, 42–44]. All variables showed significance association. The pooled Meta regression indicated that, there is association between partner fertility desire and fertility desire (OR = 16.8, 95% CI: 9.45, 29.88) and random effect model was used for this analysis (I^2^ = 85.0%, *P* = < 0.001) (Fig. 7).

#### Association between fertility desire and number of alive child

**Fig. 8:**
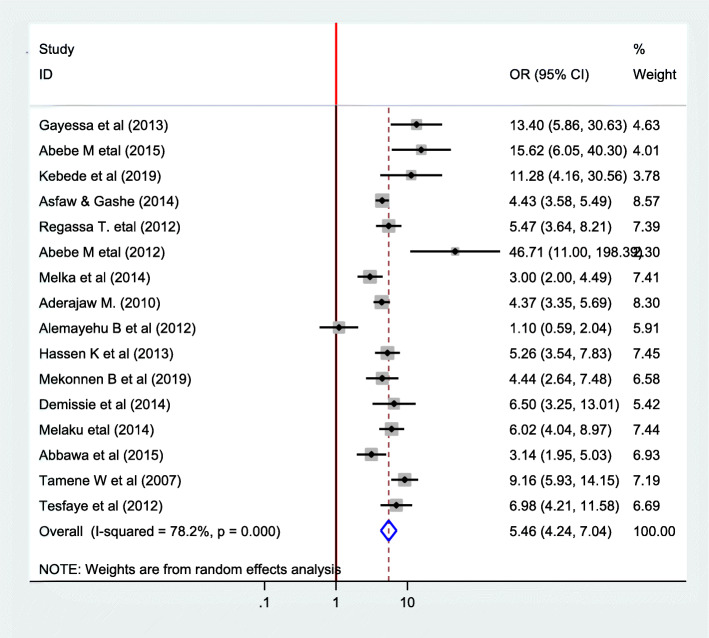
Forest plot of association between fertility desire among people with HIV positive and number of alive children in Ethiopia, 2020

In this Meta regression to find association between fertility desire and number of alive children that the parents have, sixteen articles have been included [22–29, 32, 33, 36, 39–44]. Except one article [32] all of them showed significant association. The pooled meta-regression indicated that there is significant association between fertility desire and number of alive child that the parents have. Accordingly, those who have no children have fertility desire 5.46 times than those who has one and more (OR = 5.46 95%CI 4.24, 7.040). Again in this since there is high heterogeneity random effect model was used (I^2^ = 5.46 *P* = 0.00) (Fig. 8).

#### Association between fertility desire and use of family planning

**Fig. 9:**
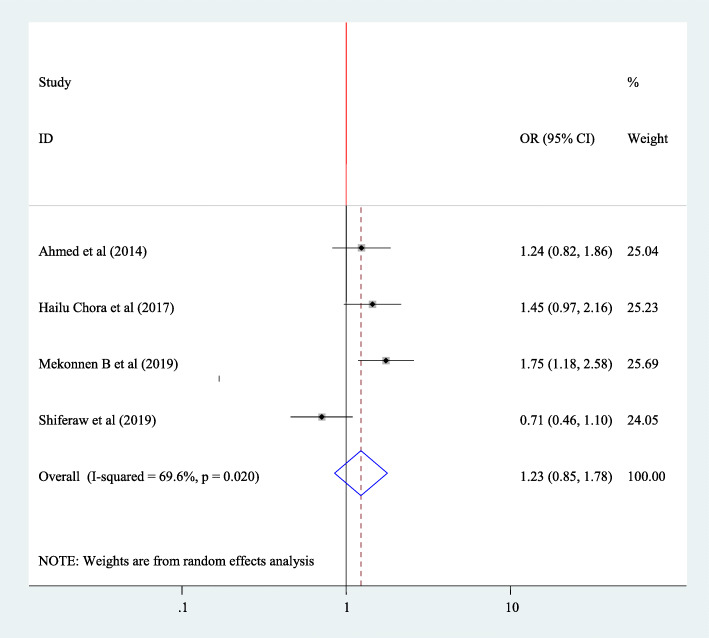
Forest plot of association between fertility desire among people with HIV positive and and use of family planning in Ethiopia, 2020

To find association between fertility desire and use of family planning four articles have been included in this study [20, 30, 36, 42]. All articles didn’t show significant association. The pooled finding of meta-analysis showed that there is no significant association between use of family planning and fertility desire (OR = 1.23 95% CI 0.85, 1.78) and random effect model was used for this regression (Fig. 9).

#### Association between fertility desire and disclosure status

**Fig. 10:**
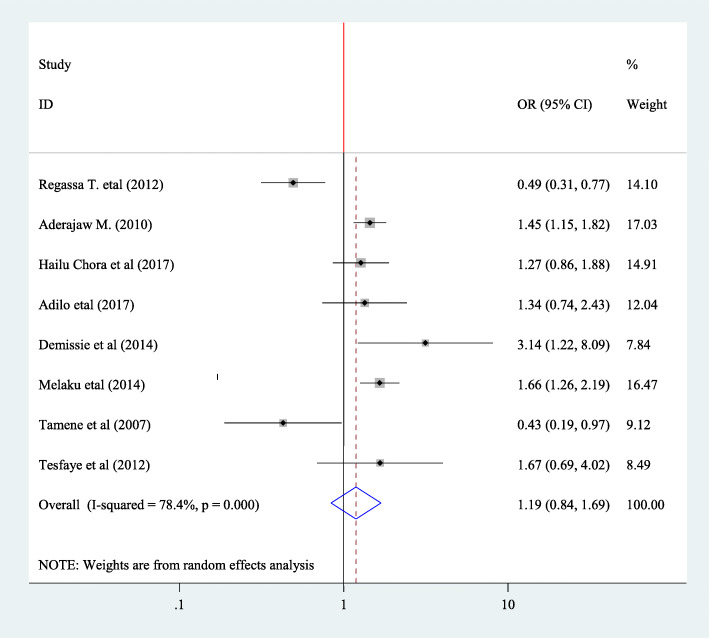
Forest plot of association between fertility desire among people with HIV positive and and disclosure status in Ethiopia, 2020

In this Meta regression, to find association between fertility desire and disclosure status, five articles have been included [20, 28, 35, 39, 40]. From these variables, only one showed significance association [39]. The pooled Meta regression indicated that, there is no association between disclosure status and fertility desire (OR = 1.10, 95% CI: 0.69, 1.75) and random effect model was used for this analysis (I^2^ = 69.3.0%, *P* = < 0.011) (Fig. 10).

#### Association between fertility desire and residence

**Fig. 11:**
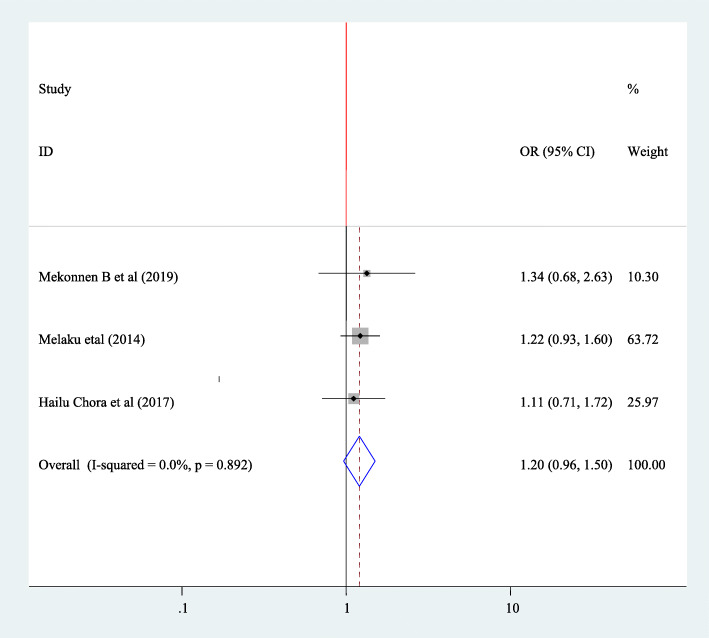
Forest plot of association between fertility desire among people with HIV positive and and residenceof participants in Ethiopia, 2020

To find the association between fertility desire and residences of the participants, three articles have been included in this study [30, 36, 40]. Nothings of those article has been showed significant association and the pooled Meta regression also showed that there is no significant association between fertility desire and residence of the participants (OR = 1.20 95% CI 0.96, 1.50). Since there is no heterogeneity fixed effect model was used in this Meta regression (I^2^ = 0.00 *P* = 0.892) (Fig. 11).

#### Association between fertility desire and knowledge of participant about PMTCT

**Fig. 12:**
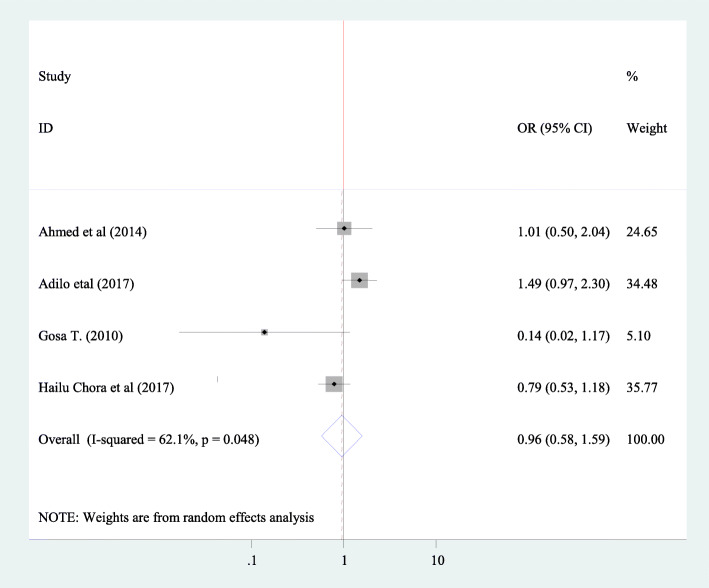
Forest plot of association between fertility desire among people with HIV positive and and knowledge of the participants about PMTCT in Ethiopia, 2020

To find association between knowledge of peoples with HIV positive about PMTCT, four articles have been included in this study [20, 30, 35, 45]. From all involved article nothing of them showed significant association. The pooled Meta regression again showed consistence indication that there is no significant association between fertility desire and knowledge about fertility in people with HIV positive (OR = 0.96955 CI 0.58, 1.59) and random effect model was used in this analysis (I^2^ = 62.1 *P* = 0.048) (Fig. 12).

#### Association between fertility desire and age

**Fig. 13:**
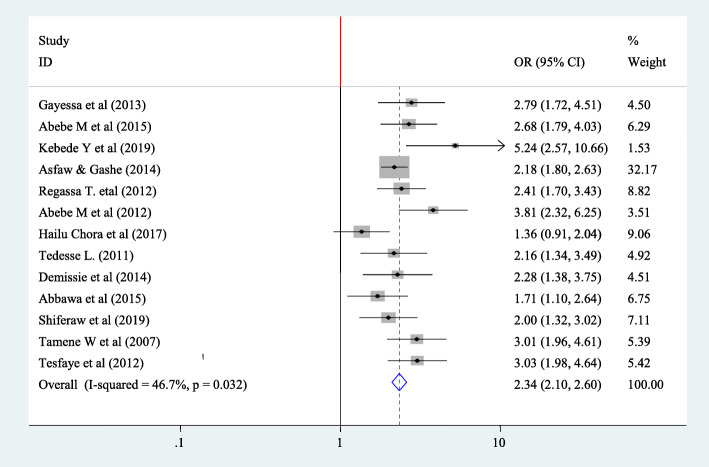
Forest plot of association between fertility desire among people with HIV positive and and age of the participants in Ethiopia, 2020

To find the fertility desire and age of the peoples with HIV positives thirteen article have been included in this study [22–27, 30, 38, 39, 41–44]. From all articles only one doesn’t show significant association [30]. The pooled Meta regression indicated that there is significant association between fertility desire and age of people with HIV. Accordingly, the pooled Meta regression indicated that those with age less than thirty have fertility desire 2.34 times more than with those aged greater than thirteen (OR = 2.34,95%CI 2.10,2.60). Since there is no more heterogeneity fixed effect model was used in this Meta regression (I^2^ = 46.7 *P* = 0.032) (Fig. 13).

#### Association between fertility desire and educational status

**Fig. 14:**
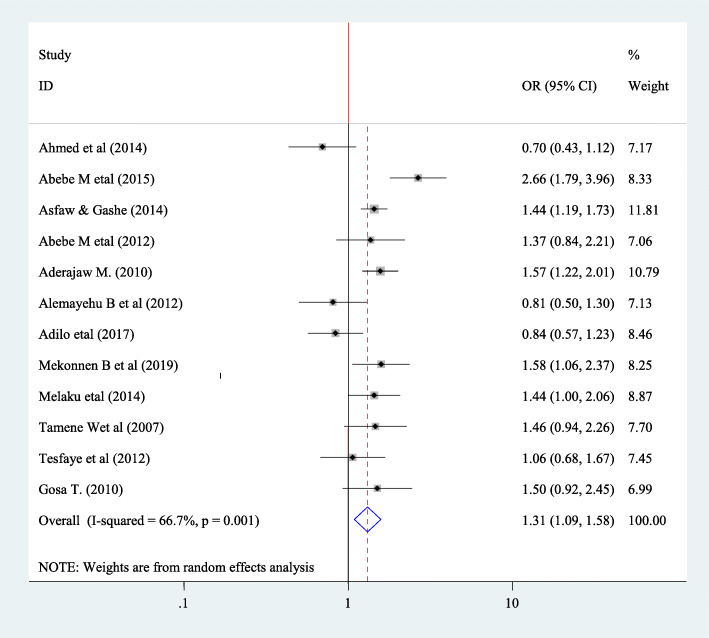
Forest plot of association between fertility desire among people with HIV positive and and educational status of the participants in Ethiopia, 2020

In this meta regression to find the association between educational status and fertility desire among HIV positive peoples twelve article have been included [20, 23, 25, 27, 29, 32, 35, 36, 40, 43–45]. From those seven of them don’t show significant association [20, 27, 32, 35, 43–45] The pooled finding indicated that there is significant association, that is these who have education have fertility desire 1.31 times than those who has no education (OR = 1.31 95%CI 1.09,1.59) and for this random effect model was used (I^2^ = 66.7%, *P* = 0.00) (Fig. 14).

## Discussion

Globally, people living with HIV specifically, women of reproductive age group remained the highest share holder of HIV/AIDS related morbidity and mortality. Nowadays peoples living with HIV/AIDS are living their sexual and reproductive life better than ever before. Similar to HIV negative peoples, PLWHA also desires to have a biological child and preserve the endurance of their descendants to the forthcoming generation. Therefore, as part of continuum HIV care, understanding fertility desire and reproductive health care needs of HIV positive women is a right thing for a right and important in planning and organizing appropriate health services.

In this systematic review and meta-analysis, we computed the pooled estimates of prevalence of fertility desire and associated factors. We found that fertility desire was high among PLHIV on ART in Ethiopia. Even though the prevalence of fertility desire differs from region to region, this study illustrated that the pooled prevalence of fertility desire among women on ART in Ethiopia is 42.21%. The difference from region to region might be attributed to the year and study population. Indeed, the relative increment from previous studies to recent one might also be attributed to improvement in health related quality of life of PLWHA than ever before.

Moreover the fertility desire among PLHIV in our study is slightly lower than the general fertility desire of the general population of Ethiopia for the 3 years preceding the survey in which 56 % of the general population wants to have another child [46]. This difference with the general population perhaps be attributed to the concern of PLWH about the vertical transmission (in which women might worry for their newborn child become infected) as evidenced by study conducted in Addis Ababa in which 4% of HIV-exposed children were positive by their 18 months [5].

The finding is comparable with findings from different countries like Brazil (39.9%) [47], Tanzania (37.1%) [14]. However, the finding is lower than findings from Canada in which 69% of the respondents want to give birth [48]. This discrepancy might be attributed to the socio-demographic characteristics and the life styles of the study populations.

This systematic review and meta-analysis also identified factors associated fertility desire among PLWHA in Ethiopia. Sex of the participants is one of the factors that had association with a fertility desire. Being female had negative association with fertility desire as compared with men. This finding is supported by a study conducted in different developing countries [12, 49–53] Men’s greater desire may be due to the fact that Men are more likely to reproduce their offspring and leave something of themselves and ancestry behind when they die. Thus it is vital that it helps policy makers and health care providers to improve partners’ involvement in decision making regarding the reproductive health service utilization in order to prevent unwanted pregnancy and mother to child transmission. And also, it might be attributed to the fact that women had less decision making power than their husbands.

Partner’s fertility desire is also another important factor that is associated with fertility desire of PLWHA. This finding is supported by evidence from Dominican Republic, in which participants who had negative perception about their partners towards pregnancy are less likely to desire pregnancy [54] Similarly, this finding is also supported by findings from South Africa [53] This finding has great policy implication in which policy makers and health care providers can capitalize on family planning and fertility related issues information should focus on couple centered.

This analysis also found that participants who had no alive child were more likely to be fertile than their counter parts. This finding is supported by review done by Berhan, Y et.al [55] and findings from Uganda [56]. Hence, according to this finding there is high probability of participants who desire child to be engaged in pregnancy risk behavior. Therefore, HIV care and treatment could be an important get way to provide information and counseling to the partners that would assure their involvement more in reproductive health services and PMTCT.

The other finding from this study is that those participants aged less than thirty were more likely to desire fertility in the future than participants aged greater than thirty. This indicates as being young is associated with likelihood of being fertile [53]. Beside this, it might be attributed the fact that majority of human being in the fertile age regardless of HIV status are more likely to have a child. Also, since young individuals are sexually active than their counterparts targeting those cohorts will play paramount role in reducing risky sexual behaviors and PMTCT. Beside this many efforts are needed to make family planning services accessible for the young females in the developing regions of the country according to their context.

This systematic review and meta-analysis also identified educational status of the participants is another important factors that determines the fertility desire of PLWHA. Those who have education are more likely to desire future fertility than their counterparts. This might be attributed to the fact that educated peoples have better decision making than individuals with no formal education. Beside this, educated peoples have better adherence status which in turn results in suppression of viral load and this makes them to regain their previous healthy life However, our finding is contrary to previous review done by Berhanet. al. which found as educational level is found to have no influence on their fertility desire [55].

### Limitation of the study

In this review all of the studies included in the current review were cross-sectional study design in nature; as a result, the outcome variable might be affected by other confounding variables.

## Conclusion

In this finding, significant people of living with HIV have a desire to have a child. Males, young age, educated and childless have strong desire for a child. The finding showed the need to strengthen fertility desire and reproductive health care needs of HIV positive peoples. People living with HIV need a great support to have safe conception and meet their fertility desire. This shows health policy should strengthen the reproductive health services of women’s living with HIV to meet their fertility needs. Therefore, strengthening the integration of fertility related issues with HIV continuum care will play a paramount role in averting risky sexual behaviors and PMTC among peoples on ART.

## Data Availability

All relevant data are within the paper and its Supporting Information files.

## Abbreviations

PLHIV: People Living with HIV
ART: Anti-retroviral Therapy
PMTCT: Prevention of Mother to Child Transmission
HIV: Human Immunodeficiency Virus
